# Post-operative opioid pain management patterns for patients who receive hip surgery

**DOI:** 10.1186/s13011-017-0094-5

**Published:** 2017-03-16

**Authors:** Chad E. Cook, Daniel I. Rhon, Brian D. Lewis, Steven Z. George

**Affiliations:** 1 Division of Physical Therapy, Duke University, 2200 West Main Street, Durham, NC 27705 USA; 2 Clinical Outcomes Research, Center for the Intrepid, Brooke Army Medical Center, Fort Sam Houston, San Antonio, TX 78234 USA; 3 Department of Orthopaedics, Duke University, Box 3389, Durham, NC 27710 USA; 4 Department of Orthopaedics, Musculoskeletal Research, Duke Clinical Research Institute, 2400 Pratt Street, Durham, NC 27705 USA; 5 Baylor University, Fort Sam Houston, San Antonio, TX 78234 USA

**Keywords:** Femoroacetabular impingement, Surgery, Opioid management

## Abstract

**Background:**

Identifying optimal, post-operative opioid management strategies is a priority of health providers and government agencies. At present, there are no studies we are aware of that have formally investigated opioid prescribing patterns for post-operative non-arthroplasty orthopedic conditions such as femoroacetabular impingement, nor has any study investigated the influence of opioid prescription patterns on health care costs and utilization. We aimed to investigate a subgrouping scheme associated with post-operative opioid prescription strategies and measure the subgroups’ direct and indirect health care utilization and costs in individuals undergoing non-arthroplasty orthopedic hip surgery.

**Methods:**

The study was an observational cohort of routine military clinical practices. We used cluster analysis to characterize pre-operative (12 months) and post-operative (24 months) opioid prescription patterns. Linear mixed effects modeling (with statistical controls for baseline status) identified opioid prescription pattern subgroups and identified subgroup differences in health care utilization and costs.

**Results:**

Two distinct clusters were identified representing 1) short-duration, high total days’ supply (SD-HD), and 2) long-duration, lesser total days’ supply (LD-LD) post-operative prescription patterns. Significantly higher costs and health care utilization for both hip-related and non-hip-related variables were consistently identified in the SD-HD group.

**Conclusions:**

Long-term opioid prescription use has been identified as a concern, but our findings demonstrate that LD-LD post-operative opioid management for hip surgery recipients was associated with lower costs and utilization. Whether these management patterns were a reflection of pre-operative health status, impacted pain-related outcomes, or can be replicated in other orthopedic procedures remains a consideration for future studies.

**Trial registration:**

NA.

## Background

In March 2016, the Centers for Disease Control and Prevention (CDC) published a series of guidelines designed to improve communication between health care practitioners and patients regarding the risks and benefits of opioid drug therapy [1]. Within the guidelines, suggestions associated with dosage, duration, follow-up, and discontinuation received emphases, specifically regarding the lowest effective dosage [2]. The CDC had been prompted to act to address the increasing number of overdose deaths and use/abuse escalations in those with non-cancer-related pain [3]. Whereas recommendations for use of a multimodal therapeutic approach that includes opioids are available for acute, general surgery peri-operative management [4], opioid management strategies for intermediate- to long-term post-surgical musculoskeletal pain have not been clearly defined. Judicious medication management, including the use of opioid therapies, is recommended [5], but prescription patterns, dosing, and timing appear to vary markedly among prescribers.

In addition, whereas immediate pain management strategies for total hip arthroplasty have been well explored peri-operatively [6], long-term strategies remains mostly uninvestigated. Non-arthroplasty-related hip surgeries are on the rise, with femoroacetabular impingement (FAI) syndrome surgery exhibiting an 18-fold increase in the United States [7]. Long-term non-arthroplasty-related pain management is mostly unexplored. To our knowledge, despite the notable escalation in surgery rates, only four studies [8–11] have discussed pain medication management strategies for non-arthroplasty orthopedic interventions such as FAI. All involved (acute) peri-operative opioid management only.

In light of the recent charge of reducing opioid use published by the CDC [1] and because of the escalating levels of non-arthroplasty hip surgeries in the United States, exploration of post-operative opioid prescription patterns is warranted. We targeted patients within the Military Health System because of existing mechanisms associated with data capture pre- and post-operatively and higher likelihood of homogeneity of care. Our primary aim was to investigate a subgrouping scheme associated with post-operative opioid prescription strategies (number of opioid prescriptions and longevity of the prescription) toward direct (hip-related) and indirect (total care) health care utilization and costs. We hypothesized that the clustered subgroup patients who received more prescriptions of opioids over longer durations would be associated with greater direct and indirect utilization and costs. Our study has potential to add to the existing literature because optimal patterns of opioid prescription have yet to be explored for many common musculoskeletal surgeries. Additionally, these findings could contribute to improving recognition of opioid prescription patterns and how these are associated with downstream costs and utilization.

## Methods

### Reporting guidelines

The study design warranted the use of the REporting of studies Conducted using Observational Routinely collected health Data (RECORD) initiative [12]. RECORD was created to improve the transparent reporting of observational studies—such as cohort, case-control, and cross-sectional studies—and recommend minimum reporting standards for observational studies [12, 13]. Ethical approval of our study was given by the Institutional Review Board at Brooke Army Medical Center.

### Study design

The study was an observational case series of routine clinical practices of Department of Defense (DOD) beneficiaries, seen in both military and civilian network clinics, who received non-arthroplasty hip-related surgery. Data were collected from July 1, 2003, through June 30, 2015, and covered 12-month pre-operative and 24-month post-operative timelines.

### Data sourcing

Data were pulled from the Military Health System Data Repository (MDR), which serves as the centralized data repository for all Defense Health Agency corporate health care data. MDR data are collected from a worldwide network of more than 260 DOD health care facilities and non-DOD entities. Data include every person-level interaction for health care, both inpatient and outpatient. MDR data are carefully processed by the Defense Health Agency, updated monthly, and available to a select group of researchers with special data usage agreements.

### Selection of variables

#### Sample

To keep the sample homogenous, we attempted to identify a specific hip surgical population, FAI syndrome, for which the number of surgeries performed has increased remarkably [7]. Since a dedicated International Classification of Diseases (ICD)-9 code for this condition does not exist, we identified surgical procedure codes most often used for this condition, excluded any surgical codes associated with arthroplasty, and then excluded any non-FAI conditions that might also receive this same surgical procedure in the 12 months before the surgery (e.g., hip osteoarthritis; avascular necrosis of the hip; hip fracture; osteomyelitis of the hip; malignant neoplasms of the pelvis, hip, or lower extremity; or other arthritic hip conditions).

### Study variables

#### Clustered variables

To better understand the pattern of opioid prescription, we a priori clustered two variables: 1) the number of days post-operative to the last opioid prescription (date of last opioid prescription – date of surgery), and 2) the days’ supply variable from MDR, which measures the number of days that the medication covers if taken as prescribed, representing the dose based on a multiple of the strength and daily frequency of each individual intake. Cluster analysis—also called segmentation or taxonomy analysis—is an explorative approach that identifies grouping structures within data [14]. Cluster analysis identifies homogenous subgroups in situations where the grouping is not previously known.

#### Descriptive variables

Age, sex, military service branch, socioeconomic status, hip surgery type (closed or open), and whether the surgery occurred in a network or military health facility were collected to describe the sample. We also examined total health care utilization in the prior 12 months, because the daily number of health care services during the prior year is related to higher use after a specific event such as surgery (service is defined as a medical visit for any health care service) [15]. Our health care utilization variables differentiated between hip-related and non-hip-related visits that were coded within the MDR.

#### Comorbidities

We identified a list of medical comorbidities within the MDR that we found to have a significant association with orthopedic injury and surgical outcomes. We captured pre-operative systemic arthropathy (osteoarthritis or other forms of systemic disease, rheumatoid or psoriatic arthritis), chronic pain, metabolic disorders, substance abuse, insomnia, mental health problems, and post-traumatic stress disorder.

#### Outcome variables

We captured variables associated with post-operative health care utilization and costs. In the MDR, costs are sub-categorized by total costs of all health care interventions (which include hip-related and non-hip-related costs) and total costs of hip-related health care interventions—each broken down by provider type. Visits were structured by provider, similarly to costs.

#### Opioid prescriptions

We expected a majority of opioid prescriptions peri-operatively (day 0 to day 3). For this study, we were interested in non-acute, post-operative opioid management strategies. Subsequently, we removed all opioid prescriptions that were provided at the index date of surgery through day 3, post-operation. All opioid prescriptions represented in the data are those that occurred from day 4 (post-operative) to month 24.

### Missing values

Data in the MDR are processed weekly. Part of the processing involves encounter validation and replacement of missing values prior to release to public consumers, researchers, or policy makers. Within the dataset, 98.11% of cases had complete data and 99.83% of values were complete. Because these missing data were marginal, we opted not to impute data.

### Data analysis

There were three main analyses used in this study. First, we used two-step cluster analysis to identify post-operative opioid use subgroupings after running pre-clustering methods. This method is designed to reveal natural subgroupings (or clusters) within a dataset that would otherwise not be apparent. Two-step cluster analysis handles scale and ordinal data and automatically selects the number of subgroups using the Bayesian information criterion [16].

Secondly, we used descriptive statistics to describe the groups that were divided by opioid use clusters and comparatively analyzed both groups using SPSS version 23.0. Nominal variables were compared using chi-square analyses. Continuous variables were evaluated using linear mixed effects modeling.

Lastly, we used linear mixed effects modeling to compare costs and utilization rates between the opioid use-based, clustered groups. Linear mixed effects modeling methods are flexible, model individual change, and accommodate for missing data (when present). We ran two analyses, unadjusted and adjusted, in which we controlled for well-represented baseline characteristics that were significantly different. For all analyses, a *p*-value < 0.05 was used to determine statistical significance.

## Results

In total, 1219 surgical recipients were included in the planned analyses. Clustering identified two distinct post-operative opioid prescription subgroups: 1) short-duration, high total days’ supply (SD-HD: *N* = 850), and 2) long-duration, less total days’ supply (LD-LD: *N* = 369). The overall trend was that the SD-HD subgroup had more pre-operative comorbidities, opioid prescriptions, and total days of opioid pain medications. That subgroup was also younger and had higher proportions of heavy pre-operative health care utilization. Baseline differences among clustered groups are reported in Table 1.

**Table 1:** Comparative analyses between clustered groups (baseline) *N* = 1219

Variable	Group 1 short-term duration, higher total days’ supply *N* = 850	Group 2 long-term duration, lower total days’ supply *N* = 369	*P*-value
^a^Pre-op opioid prescription	765 = yes	351 = yes	**<0.01**
	72 = no	14 = no	
	13 = missing	4 = missing	
^a^Sex	471 = male	213 = male	0.46
	379 = female	156 = female	
Age	31.38 (7.83)	32.44 (7.98)	**0.03**
^a^Military service branch	389 = Army	149 = Army	0.07
	13 = Coast Guard	5 = Coast Guard	
	197 = Air Force	110 = Air Force	
	110 = Marines	35 = Marines	
	139 = Navy	68 = Navy	
	2 = Other	2 = Other	
^a^Officer/enlisted	687 = enlisted	346 = enlisted	**0.04**
	163 = officer	23 = officer	
^a^Hip surgery type	767 = closed	430 = closed	0.64
	83 = open	26 = open	
Network or MHS	475 = MHS	194 = MHS	0.28
	375 = network	175 = network	
Number of opioid prescriptions			
Pre-op	3.19 (4.43)	2.18 (1.85)	**<0.01**
Post-op	7.39 (2.21)	2.21 (1.95)	**<0.01**
Last recorded opioid prescription (days from surgery)	38.71 (52.91)	406.24 (152.61)	**<0.01**
^a^Arthropathy (pre-op)	573 = no	243 = no	0.59
	277 = yes	126 = yes	
^a^Metabolic disorders (pre-op)	754 = no	344 = no	**0.02**
	96 = yes	25 = yes	
^a^Substance abuse (pre-op)	707 = no	319 = no	0.15
	143 = yes	50 = yes	
^a^Cardiac problems (pre-op)	784 = no	339 = no	0.83
	66 = yes	30 = yes	
^a^Insomnia (pre-op)	794 = no	352 = no	0.26
	53 = yes	17 = yes	
^a^Heavy pre-op health utilizers	380 = no	212 = no	**<0.01**
	467 = yes	157 = yes	

^a^Represents use of chi-square analyses. All continuous analyses used linear mixed effects modeling

**Fig. 1:**
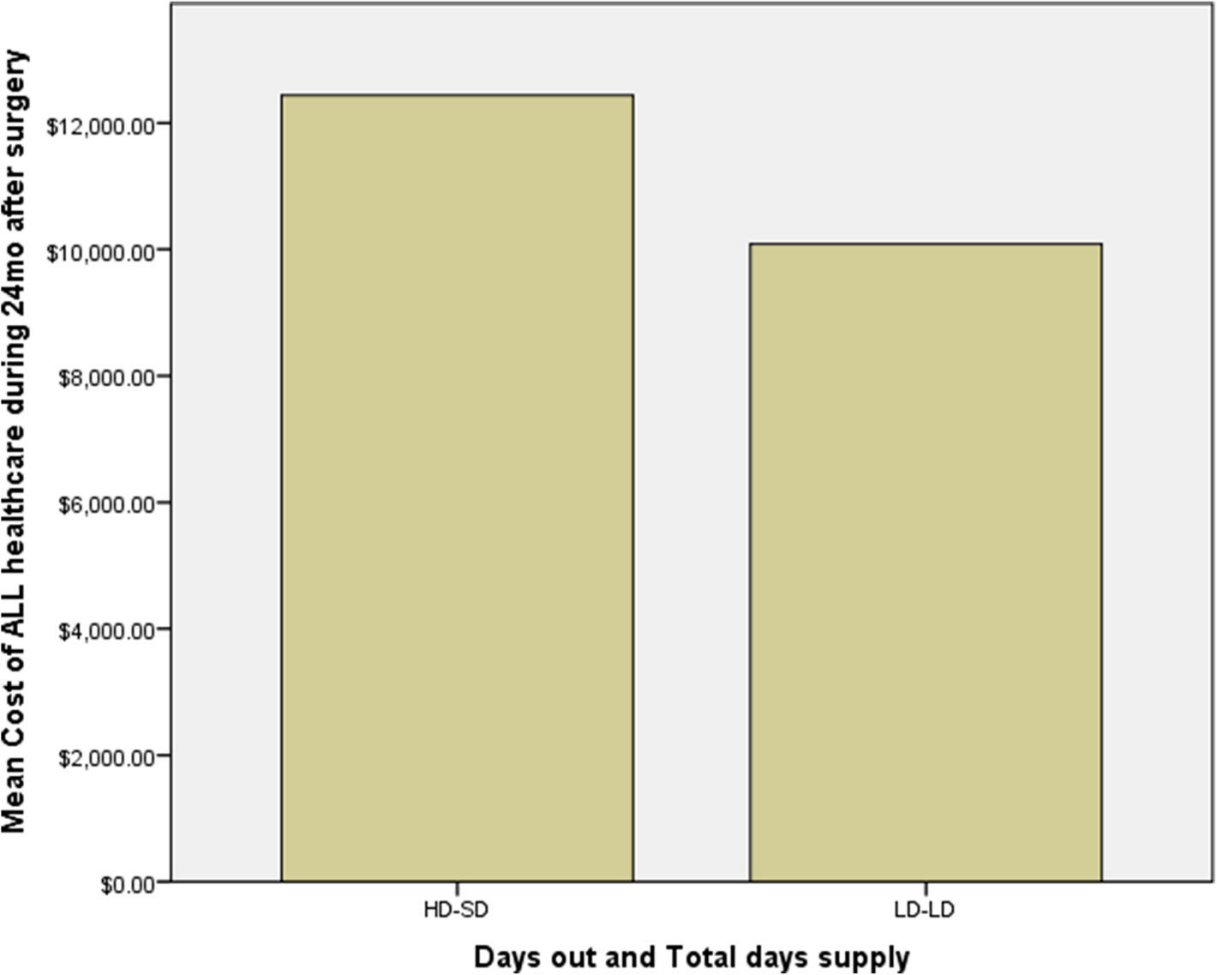
*Bar graph* representing unadjusted total costs of care divided by subgroups of opioid prescription

**Fig. 2:**
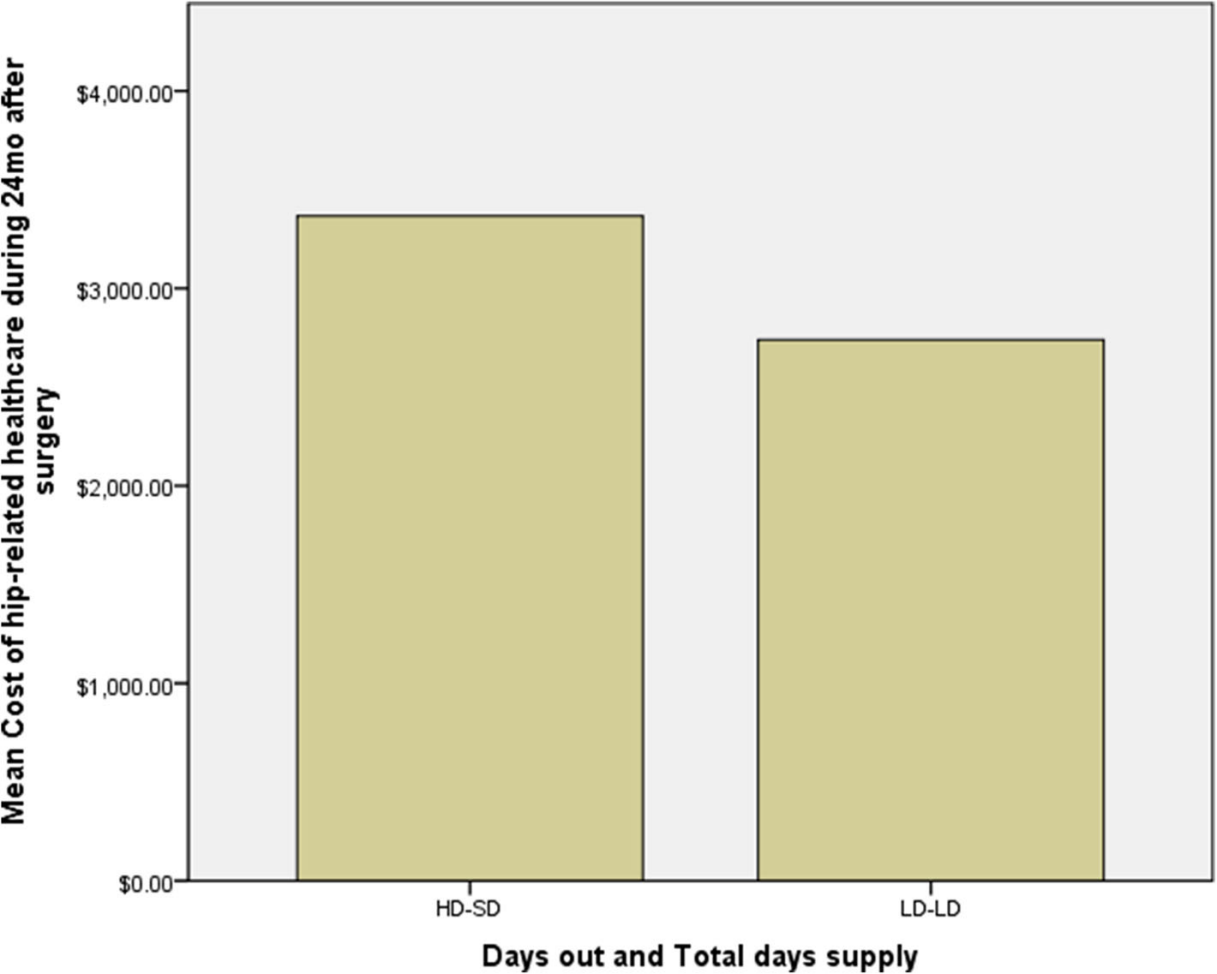
*Bar graph* representing unadjusted total hip-related costs of care divided by subgroups of opioid prescription

Table 2 involves linear mixed effects modeling and outlines unadjusted differences in costs and health care utilization. Statistically significant between-subgroup differences were noted with total health care visits and costs as well as total hip-related health care visits and costs—all indicating higher costs and utilization in individuals with SD-HD. Those in the SD-HD group also had significantly higher total days’ supply of *all* pain-related medications. Fig. 1 provides a graphical representation of all healthcare costs by group comparison whereas Fig. 2 represents the differences in hip related costs.

**Table 2:** Unadjusted bivariate analyses of post-operative outcomes between clustered groups

Variable	Degrees of freedom	Group 1 short-term duration, higher total days’ supply *N* = 850	Group 2 long-term duration, lower total days’ supply *N* = 369	*P*-value
Total health care visits (post-op)	1217	96.99 (68.26)	76.99 (54.17)	**<0.01**
Total health care costs (post-op)	1217	$31,944.44 ($27,473.44)	$27,765.60 ($33,075.09)	**0.02**
Total hip-related visits (post-op)	1217	32.87 (28.90)	25.12 (23.49)	**<0.01**
Total hip-related costs (post-op)	1217	$16,888.09 ($15,522.37)	$14,153.19 ($18,670.25)	**0.02**
Total days’ supply of all pain-related medications	1217	1148.00 (1138.01)	885.34 (927.60)	**<0.01**
Cost of pain meds (post-op)	1217	$859.30 ($3682.92)	$680.55 ($3853.24)	0.44

Bolded *p*-values reflect statistical significance <0.05. All analyses involve linear mixed effects modeling

Table 3 involves linear mixed effects modeling and reflects adjusted differences in costs and health care utilization, using the controls of age and pre-operative opioid use, pre-operative metabolic syndrome, and pre-operative health utilization behaviors. Findings were similar to the unadjusted totals, with between-subgroup differences in total days’ supply of pain medications, total health care visits and costs, and total hip-related health care visits and costs—all indicating significantly higher costs and utilization in individuals with SD-HD.

**Table 3:** Adjusted bivariate analyses of post-operative outcomes between clustered groups

Variable	Degrees of freedom	Group 1 short-term duration, higher total days’ supply *N* = 850	Group 2 long-term duration, lower total days’ supply *N* = 369	*P*-value
Total health care visits (post-op)	1202	97.51 (68.44)	76.88 (54.09)	**<0.01**
Total health care costs (post-op)	1202	13,155.48 (9369.88)	10,721.88 (10,570.18)	**<0.01**
Total hip-related visits (post-op)	1202	32.94 (28.93)	25.26 (23.57)	**<0.01**
Total hip-related costs (post-op)	1200	$16,891.10 ($15,581.68)	$14,575.15 ($18,737.44)	**0.03**
Total days’ supply of all pain-related medications	1200	1,157.52 (1,143.02)	891.36 (930.34)	**<0.01**
Cost of pain meds (post-op)	1202	$871.15 ($3,710.12)	$679.09 ($3,871.75)	0.42

Bolded *p*-values reflect statistical significance <0.05. Includes linear mixed effects modeling with control for age and pre-operative opioid use, pre-operative metabolic syndrome, and pre-operative health utilization behaviors

## Discussion

Our study investigated an empirically determined subgrouping scheme based on post-operative opioid prescription strategies and compared the derived subgroups on direct (hip-related) and indirect (total care) health care utilization and costs. We targeted individuals seeking care in the Military Health System who had undergone non-arthroplasty orthopedic hip surgery in hopes of homogenizing the patient population and care processes. Furthermore, the Military Health System is the closest example of a single-payer system in the United States, which provides an additional advantage when analyzing care-related trends. Rather than attempt to authoritatively define subgroups, cluster analysis identified distinct post-operative prescription patterns as 1) SD-HD and 2) LD-LD. Patient characteristics, costs, and health care utilization were different depending on group allocation, and there are a number of potential reasons for these findings.

In the literature, we found no data on post-operative opioid prescription patterns for non-arthroplasty orthopedic hip surgical management and only limited information overall on strategies of opioid prescription patterns for *any* form of surgery. For our study, we used Bayesian information criteria to define homogenous subgroups of opioid prescriptions, and two strong, well-defined, distinct patterns emerged. In this cohort, those in the SD-HD subgroup exhibited higher overall health care costs and utilization, whether it was related to the hip or not. In our initial hypothesis, we assumed that longer-term post-operative use would be grouped together with higher doses of opioid prescriptions, but this was not the case when subgroups were determined empirically. Longer-term use was related to fewer overall prescriptions and days’ supply of opioids; and conversely, shorter-term use was related to more prescriptions and days’ total supply. We consider four propositions on the unique patterns that were robustly identified by the subgroups.

### Proposition one

We suggest that the post-operative opioid prescription patterns observed are reflective of pre-operative comorbid conditions. It is well documented that sociodemographic factors, pain, prior drug use, genetics, environmental factors, psychosocial problems, and established addictive behaviors (e.g., alcohol abuse) increase the risk of opioid drug use [17]. Concomitant psychosocial problems such as chronic pain syndrome, depression, anxiety, and other forms of social phobia, especially at a younger age, are also stronger predictors of opioid use and abuse [18]. Within our study, those in the SD-HD subgroup had higher proportions of pre-operative mental health diagnoses and substance abuse histories and were younger on average. The SD-HD prescription pattern observed in this cohort may reflect a management process in which health care provider prescription pattern was reactionary to those conditions.

### Proposition two

The SD-HD subgroup was likely associated with the pre-operative opioid use patterns of its patients. Generalized risks of opioid misuse, abuse, dependence, or addiction are well studied; and prior opioid use also leads to an increase in tolerance of opioids (or a state of adaptation) [18]. Prior exposure to an opioid can result in a diminution of one or more opioid effects over time and reduces the clinical effect of the prescription [19]. When analyzing our pre-operative data, we found that those in the SD-HD subgroup had 1.01 more prescriptions (on average) and 15.56 more total days of opioid medication than those in the LD-LD subgroup. This proposition is consistent with others who have suggested the risk of persistent post-operative pain after different forms of surgery is associated with higher levels of pre-operative opioid use [20, 21].

### Proposition three

We hypothesize that those in the LD-LD subgroup may have benefitted more from opioids by receiving adequate pain management, requiring fewer prescriptions over time. Whereas the data regarding the number of pills or strength of dosage related to a prescription were unavailable, we did have data on total days of medication, which is a proxy measure that represents the number of days that the medication covers if taken as prescribed (thus representing the dose based on a multiple of the strength and daily frequency of each individual intake). This interpretation is consistent with our clinical experience and matches the decreased total pain medication days seen in the LD-LD group. This theory deserves further exploration as the idea of greater benefit from longer-term prescriptions is a paradox given the current climate for limiting opioid prescription. For example, on average, LD-LD subgroup patients received opioid prescriptions 400+ days post-operatively; yet, they had lower overall health care utilization and costs.

### Proposition four

We hypothesize that the higher costs and utilization are associated with increased incidences of side effects in the SD-HD subgroup. Previous work supports that costs associated with opioid-related constipation for non-cancer-related opioid recipients was nearly double that of non-opioid-related constipation, and that inpatient hospitalization odds were 2.3 times higher in those with constipation than in those without [22]. Other side effects such as sedation, dizziness, nausea, vomiting, physical dependence, tolerance, and respiratory depression may also lead to increased health care costs [23]. Unfortunately, our dataset does not provide data on the most common side effects or the temporal relationship of these side effects to opioid utilization.

What do these findings mean for those prescribing and receiving opioid medications post-operatively? We argue that opioid prescription strategies require further investigation into whether these subgroups replicate in other conditions outside of hip surgery and outside a single-payer system such as the Military Health System. In addition, future work should determine whether these subgroups are related to adequate patient report of pain relief, habituation, or addiction. If these subgroups are represented outside the Military Health System and in other conditions, further exploration is needed to explore why the LD-LD group exhibited less health care utilization and had lower costs.

Further, the relationship between true pain relief of the groups should be explored to determination the association of relief to lower utilization and costs. This is especially important since managing long-term opioid prescribing is a targeted objective of the CDC [1] and the American Society of Interventional Pain Physicians (ASIPP) [24], which has specifically targeted cases involving >90 days. Further, costs and health care utilization associated with opioid non-abusers may shed further light on care patterns since previous work [25] has shown that those who take opioids (at least one opioid prescription) but do not abuse them incur $1800 more in costs versus those who receive no opioid prescriptions. Our findings are similar and suggest that those who receive SD-HD will also utilize hip-related and non-hip-related health care at a higher rate, with corresponding increases in costs.

### Limitations

There are several limitations to this study. First, despite our ability to incorporate pre-operative factors and capture patterns over a 24-month period, the study design did not permit cause-and-effect statements to be made about the identified subgroups. Second, the MDR provided no specifics on the type of opioid action (short- or long-acting). Third, the MDR lacks patient-reported outcomes; thus, we are unable to determine which opioid strategy was related to better pain relief and health-related outcomes. Fourth, measures such as chronic pain may fail to represent fully all those with recognized chronic pain conditions. Lastly, the information is limited to individuals within the military payment system and may not be generalizable to other health care environments.

## Conclusions

Long-term opioid prescription use has been identified as a concern, but our findings demonstrate that LD-LD post-operative opioid management for hip surgery recipients was associated with lower costs and utilization. These results suggest that opioid management strategies for other common post-operative orthopedic procedures should be investigated in a controlled fashion to determine whether this subgroup pattern can be replicated and to better explore the effectiveness of prescription pattern on providing post-operative pain relief without excessive side effects or fostering opioid abuse. Whether these management patterns were a reflection of pre-operative health status, were affected by pain-related outcomes, or can be replicated in other orthopedic procedures remains a necessary consideration for future studies.

## Abbreviations

ASIPP: American Society of Interventional Pain Physicians
CDC: Centers for Disease Control and Prevention
DOD: Department of Defense
FAI: Femoroacetabular impingement
ICD: International Classification of Diseases
LD-LD: Long-duration, lesser total days’ supply
MDR: Military Health System Data Repository
SD-HD: Short-duration, high total days’ supply
